# The development of optic neuropathy after chronic rhinosinusitis: A population-based cohort study

**DOI:** 10.1371/journal.pone.0220286

**Published:** 2019-08-07

**Authors:** Chan-Wei Nien, Chia-Yi Lee, Pei-Hsuan Wu, Hung-Chi Chen, Jessie Chao-Yun Chi, Chi-Chin Sun, Jing-Yang Huang, Hung-Yu Lin, Shun-Fa Yang

**Affiliations:** 1 Institute of Medicine, Chung Shan Medical University, Taichung, Taiwan; 2 Department of Ophthalmology, Show Chwan Memorial Hospital, Changhua, Taiwan; 3 Department of Optometry, College of Medicine and Life Science, Chung Hwa University of Medical Technology, Tainan, Taiwan; 4 Department of Otolaryngology–Head and Neck Surgery, Tri-Service General Hospital, Taipei, Taiwan; 5 Department of Ophthalmology, Chang Gung Memorial Hospital, Linkou, Taiwan; 6 Department of Medicine, Chang Gung University College of Medicine, Taoyuan, Taiwan; 7 Center for Tissue Engineering, Chang Gung Memorial Hospital, Linkou, Taiwan; 8 Department of Otorhinolaryngology Head and Neck Surgery, Taichung Hospital, Ministry of Health and Welfare, Taichung, Taiwan; 9 Department of Ophthalmology, Chang Gung Memorial Hospital, Keelung, Taiwan; 10 Department of Chinese Medicine, Chang Gung University, Taoyuan City, Taiwan; 11 Department of Medical Research, Chung Shan Medical University Hospital, Taichung, Taiwan; 12 Department of Optometry, Chung Shan Medical University, Taichung, Taiwan; 13 Department of Exercise and Health Promotion, Chung Chou University of Science and Technology, Changhua, Taiwan; Stanford University School of Medicine, UNITED STATES

## Abstract

**Background:**

To evaluate the risk of developing optic neuropathy (ON) in patient with both non-surgery and surgery-indicated chronic rhinosinusitis (CRS) via the national health insurance research database in Taiwan.

**Methodology/Principal findings:**

44,176 Patients with a diagnostic code of CRS was selected, which included 6,678 received functional endoscopic sinus surgery (FESS) regarded as the surgery-indicated CRS. Each individual in the study group was matched to two non-CRS patients by age and gender. The outcome was set as the occurrence of ON according to the diagnostic codes occurred after the index date. Poisson regression was used to calculate the adjusted relative risk (aRR) and conditional Cox proportional model was used to estimate the adjusted hazard ratio (aHR). There were 131 and 144 events of ON occurred in the study group and the control group respectively during the follow-up period. The whole study group, whether received FESS or not, demonstrated both significant aRR and aHR compared to the control group after adjusting demographic data, prominent ocular diseases, and systemic co-morbidities. In addition, both the aRR and aHR were higher in CRS patient received FESS than those with CRS but without FESS management.

**Conclusion:**

The existence of CRS, especially the surgery-indicated CRS is a significant risk factor for the following ON using multivariable analysis.

## Introduction

Chronic rhinosinusitis (CRS) is an inflammatory disease in the paranasal sinuses that persisted at least 12 weeks, [1] and affect a major amount of population.[2] The clinical presentations of CRS include nasal stiffness, nasal discharge, facial pain, reduction of smell, headache and shortness of breath.[1, 3] In the severe form, the infection and inflammation of the paranasal sinus in CRS may even lead to the development of dread intracranial infection.[4] On the other hand, cranial nerve disorders like the dysfunction of trigeminal nerve and oculomotor nerve may occur in patient with CRS.[5–7]

Both medical and surgical managements have been utilized to treat CRS.[8] The topical corticosteroid therapy, oral corticosteroid administration and antibiotic treatment with macrolide have been applied to treat CRS with acceptable outcome.[3, 8] Functional endoscopic sinus surgery (FESS) is a well-established intervention for the severe CRS which shows poor response to medical management.[9–11] Still, the recovery of maxillary sinus mucosa in patient with CRS is incomplete one year after the FESS management.[3] Moreover, patients with some risk factors like higher Lund-Mackay CT scores and fungal-induced CRS may still experience poor quality of life or persistent nasal polyp formation even after successful FESS intervention.[12, 13] The above lines of evidence suggest that the effect of severe CRS would persist despite the performance of FESS.

In the ophthalmic disorders, orbital cellulitis and dacryocystitis have been reported in patients with previous CRS despite treatment program.[14, 15] Concerning the optic nerve, swelling of optic nerve head after CRS was observed in a previous case report.[16] In addition, the occurrence of optic neuropathy (ON) was also found in those patients with CRS whether received FESS or not.[17–19] Since the damage of optic nerve in patient with CRS is not uncommon, there may be causal relationship between the development of ON and the existence of CRS which has rarely been elucidated before.

The aim of the current study was to evaluate the possibility of developing ON in patients with CRS, including those surgical-indicated CRS patients who received FESS management, via the national health insurance research database (NHIRD) in Taiwan. Besides, several potential risk factors of ON will also be discussed and analyzed in the multivariable model.

## Material and methods

### Data source

This retrospective population-based cohort study was approved by the National Health Insurance Administration and the Institutional Review Board (IRB) of Chung Shan Medical University (number: CS-17075). All data were fully anonymized and the IRB waived the requirement for informed consent. Provided by the Taiwan National Health Research Institutes, the NHIRD contains data of insurance claims from more than 99% of Taiwan’s population. The claims data were obtained from the Longitudinal Health Insurance Database 2005 version (LHID 2005) in the current study which derived from the 2016 version of NHIRD. The LHID 2005 contains data on two million patients randomly sampled from the NHIRD registry for the year 2005. The LHID 2005 data were linked from 1 January 2000, to 31 December 2016, and both the International Classification of Diseases, Ninth Revision (ICD-9) and International Classification of Diseases, Tenth Revision (ICD-10) were used for disease diagnosis. Details on the medications prescribed for the patients and the demographics, socioeconomic status, and residence of the patients are also available in the NHIRD.

### Patient selection

Patients were defined as having CRS by the diagnosis of ICD-9 codes: 473.x, ICD-10 codes: J32.x with an otorhinolaryngologist (department code: 09) from 2000 and 2016. The index date for CRS patients was the first date of CRS diagnosis. We further identified the surgery-indicated CRS if (1) their medical records indicated the arrangement of FESS (procedure codes: 65063B and 65064B) within two year after the diagnosis of CRS, (2) the usage of corticosteroid or antibiotic for at least two years from the diagnosis of CRS. After the exclusion of index date before 2005, the newly diagnosed CRS patients were included in this study. To more accurately elucidate the association between CRS and ON, the following exclusion criteria were applied to exclude certain impaired ocular conditions: (1) receipt of a diagnosis of legal blindness (ICD-9 codes: 369.4, ICD-10 codes: H54.0x, H54.1x, H54.4x, H54.8) at any time; (2) receipt a diagnosis of ocular tumors (ICD-9 codes: 190.0–190.9, ICD-10 codes: C69.x) at any time; (3) receipt a diagnosis of severe ocular trauma (ICD-9 codes: 871.0–871.2, 871.4–871.9, ICD-10 codes: S05.2x-S05.6x) at any time; (4) receipt of any type of eyeball removal surgery or diagnosed as anophthalmos (ICD-9 codes: 16.3x, 16.4x, 16.5x, 871.3, ICD-10 codes: Q11.1, S05.7x, Z90.01 plus procedure codes: 85001C, 85002C, 86808B) before the index date; and (5) receipt a diagnosis of any type of ON (ICD-9 codes: 377.x, ICD-10 codes: H46.x, H47.x) before the index date. In addition, each individual in the study group was age and gender-matched with two non-CRS individuals (all controls were non-duplicate), as discussed in the following sections, which constituted the control group. Index date of the control group corresponded with the matched CRS patients. Furthermore, exclusion criteria for the study group was also applied to the control group. Patients with CRS who could not be matched with two non-CRS patients were excluded.

### Main outcome measurement

The development of ON was regarded as the main outcome in the current study which was based on the emergence of ON-related diagnostic codes (ICD-9 codes: 377.00–377.03, 377.10–377.12, 377.30–377.32, 377.39, 377.41, ICD-10 codes: H46.0x, H46.1x, H46.8, H46.9, H47.01x, H47.10-H47.13, H47.20, H47.21x, H47.29x) after the index date. Those ON-related diagnostic codes that indicate clearly underlying etiology (e.g. hereditary optic atrophy and toxic optic neuropathy) or involvement of other parts of visual pathway (e.g. disorders of optic chiasm) were eliminated to prevent overestimation and confusion. Furthermore, only patients who received the abovementioned diagnostic codes by an ophthalmologist (department code: 10) before 2016 from the index date were considered as having achieved an outcome and were included in the study.

### Demographic variables and co-morbidities

In the multivariable analysis (In the Poisson regression and the Cox proportional-hazards model), we adjust for health conditions, demographic conditions, and following systemic co-morbidities: hypertension, diabetes mellitus, ischemic heart diseases, hyperlipidemia, congestive heart failure, cerebrovascular disease, dementia, chronic pulmonary disease, rheumatic disease, and hemiplegia or paraplegia. To further make the ocular condition more homogenous, glaucoma, retinal vessel occlusion, age-related macular degeneration (AMD), and posterior as well as pan-uveitis were also considered in the multivariable model. The diagnostic codes of all the co-morbidities mentioned above were presented in S1 Table.

### Statistical analysis

SAS version 9.4 (SAS Institute Inc, NC, USA) was used for all analyses. After age and sex-matching at 1:2 ratio of the study and control groups, we longitudinally traced the data from the index date in a right-censored outcome: until the date of ON diagnosis, withdrawal from the National Health Insurance program, or 31 December 2016. Except the gross comparison between the study group (CRS population) and the control group, the study group was further divided into those with FESS and those without FESS. Analyses mentioned in the following sections were applied to evaluate the risk of ON among those groups.

The incidence rate, crude relative risk (RR) and corresponding 95% confidential interval (CI) were calculated using Poisson regression. In order to conduct the multivariable Poisson regression analysis, we calculated the total count of new-onset ON and offset (person-months) that was stratified by CRS exposure, sex, age group, and systemic co-morbidities at baseline. The PROC GENMOD (S2 Table) was used to estimate the crude RR of the study group in the Poisson regression, and the adjusted relative risk (aRR, the reference was control groups) of ON among CRS with FESS, CRS without FESS in the multivariable Poisson model. The setting included the Poisson distribution of count of ON as independent, log link function, and offset was the log of person-months, the dependent variables in the multivariable Poisson model including CRS exposure, sex, age group, and systemic as well as ocular co-morbidities.

In the time-to-event analysis, we performed the log-log plot to evaluate the variation of proportional-hazards which according to follow up interval, and we found the proportional-hazards were under assumption (S1 Fig). Then, the conditional Cox proportional-hazards model was adopted to compute both the crude and adjusted hazard ratios (aHR) of ON among CRS with FESS, CRS without FESS and control groups by consideration of co-variates including the aforementioned demographic data, prominent ocular diseases, and systemic comorbidities. Moreover, the aHR of ON is estimated by stratifying gender and age in subgroup analysis. We estimated Kaplan-Meier to indicate the cumulative incidence proportion of ON between the CRS patients with FESS, CRS patients without FESS and control groups, and used the log rank test to determine the significant difference between the survival curves between the study group and the control group.

The stepwise model selection (Selection of entry = 0.1, Selection of stay = 0.08, choose = Akaike information criterion) was also used to fit both the multivariable Poisson model and conditional Cox proportional-hazards model (the co-variates in this stage including hyperlipidemia, heart failure, dementia, chronic pulmonary diseases, and glaucoma at baseline) to improve accuracy. Both the conditional Cox proportional-hazards model was not adjusted for the race because most patients are Taiwanese. Statistical significance was set at a P value lesser than 0.05 and P value lesser than 0.0001 was depicted as P<0.0001.

## Results

**Fig 1:**
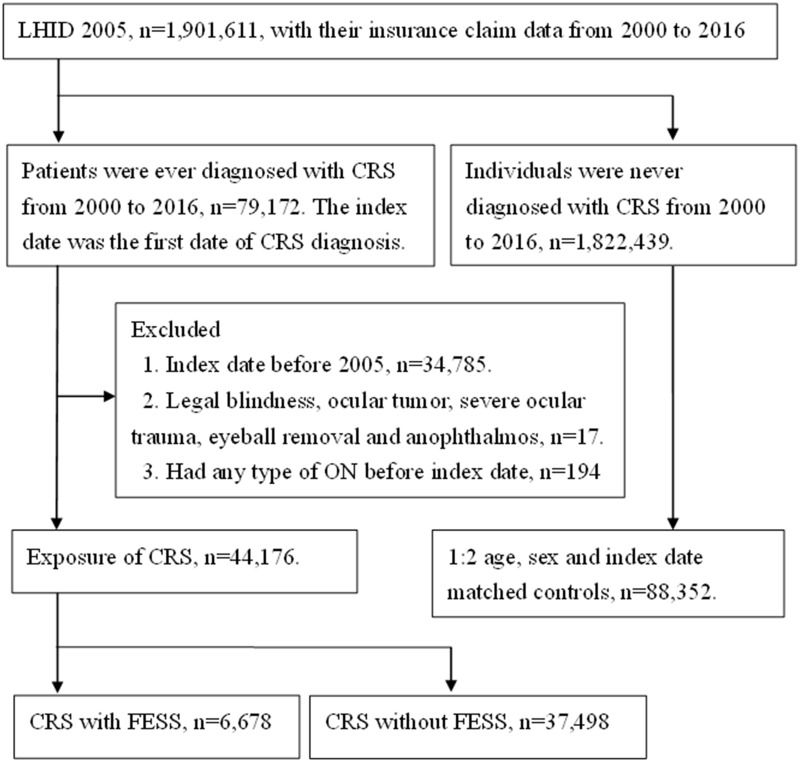
The flowchart of patient selection. LHID 2005: Longitudinal Health Insurance Database 2005 version, CRS: chronic rhinosinusitis, FESS: Functional endoscopic sinus surgery, ON = optic neuropathy.

A total numbers of 44,176 patients with CRS (included 6,678 treated with FESS within two years) were identified after exclusion, while another 88,352 individuals were age-sex matched in control group. The flowchart of patient selection is shown in Fig 1. The age and gender ratio are identical due to the matching process, while the different characteristics of co-morbidities between the study and control group are listed in Table 1.

**Table 1:** Baseline characteristics between the study and control groups.

	Control n = 88,352	CRS n = 44,176	CRS without FESS n = 37,498	CRS with FESS n = 6,678	P1	P2
Age					1.0000	<0.0001*
<40	38068(43.09%)	19034(43.09%)	16624(44.33%)	2410(36.09%)		
40–59	30576(34.61%)	15288(34.61%)	12399(33.07%)	2889(43.26%)		
60–79	17288(19.57%)	8644(19.57%)	7333(19.56%)	1311(19.63%)		
>= 80	2420(2.74%)	1210(2.74%)	1142(3.05%)	68(1.02%)		
Sex					1.0000	<0.0001*
Male	45836(51.88%)	22918(51.88%)	18793(50.12%)	4125(61.77%)		
Female	42516(48.12%)	21258(48.12%)	18705(49.88%)	2553(38.23%)		
Co-morbidities†						
Hypertension	19907(22.53%)	11735(26.56%)	9962(26.57%)	1773(26.55%)	<0.0001*	0.9770
Diabetes mellitus	10108(11.44%)	5919(13.4%)	5017(13.38%)	902(13.51%)	<0.0001*	0.7778
Ischemic heart disease	7346(8.31%)	5145(11.65%)	4428(11.81%)	717(10.74%)	<0.0001*	0.0119*
Hyperlipidemia	16035(18.15%)	10327(23.38%)	8716(23.24%)	1611(24.12%)	<0.0001*	0.1174
Heart failure	2934(3.32%)	2056(4.65%)	1803(4.81%)	253(3.79%)	<0.0001*	0.0003
Cerebrovascular disease	5526(6.25%)	3797(8.6%)	3323(8.86%)	474(7.1%)	<0.0001*	<0.0001*
Dementia	905(1.02%)	492(1.11%)	455(1.21%)	37(0.55%)	0.1330	<0.0001*
Chronic pulmonary diseases	16493(18.67%)	15921(36.04%)	14066(37.51%)	1855(27.78%)	<0.0001*	<0.0001*
Rheumatic disease	1604(1.82%)	1370(3.10%)	1178(3.14%)	192(2.88%)	<0.0001*	0.2473
Hemiplegia or paraplegia	841(0.95%)	485(1.10%)	445(1.19%)	40(0.6%)	0.0118*	<0.0001*
Glaucoma	6297(7.13%)	4429(10.03%)	3808(10.16%)	621(9.3%)	<0.0001*	0.0319*
AMD	673(0.76%)	451(1.02%)	373(0.99%)	78(1.17%)	<0.0001*	0.1943
Retinal vessel occlusion	199(0.23%)	129(0.29%)	113(0.3%)	16(0.24%)	0.0211*	0.3889
Posterior and pan-uveitis	84(0.1%)	68(0.15%)	54(0.14%)	14(0.21%)	0.0028*	0.2075

CRS: chronic rhinosinusitis

FESS: Functional endoscopic sinus surgery

AMD = age-related macular degeneration

^†^ The co-morbidities were identified before index date

P1 indicate the difference of each characters between control and CRS group

P2 indicate the difference of each characters between CRS without FESS subgroup and CRS with FESS subgroup

* denotes significant difference

**Fig 2:**
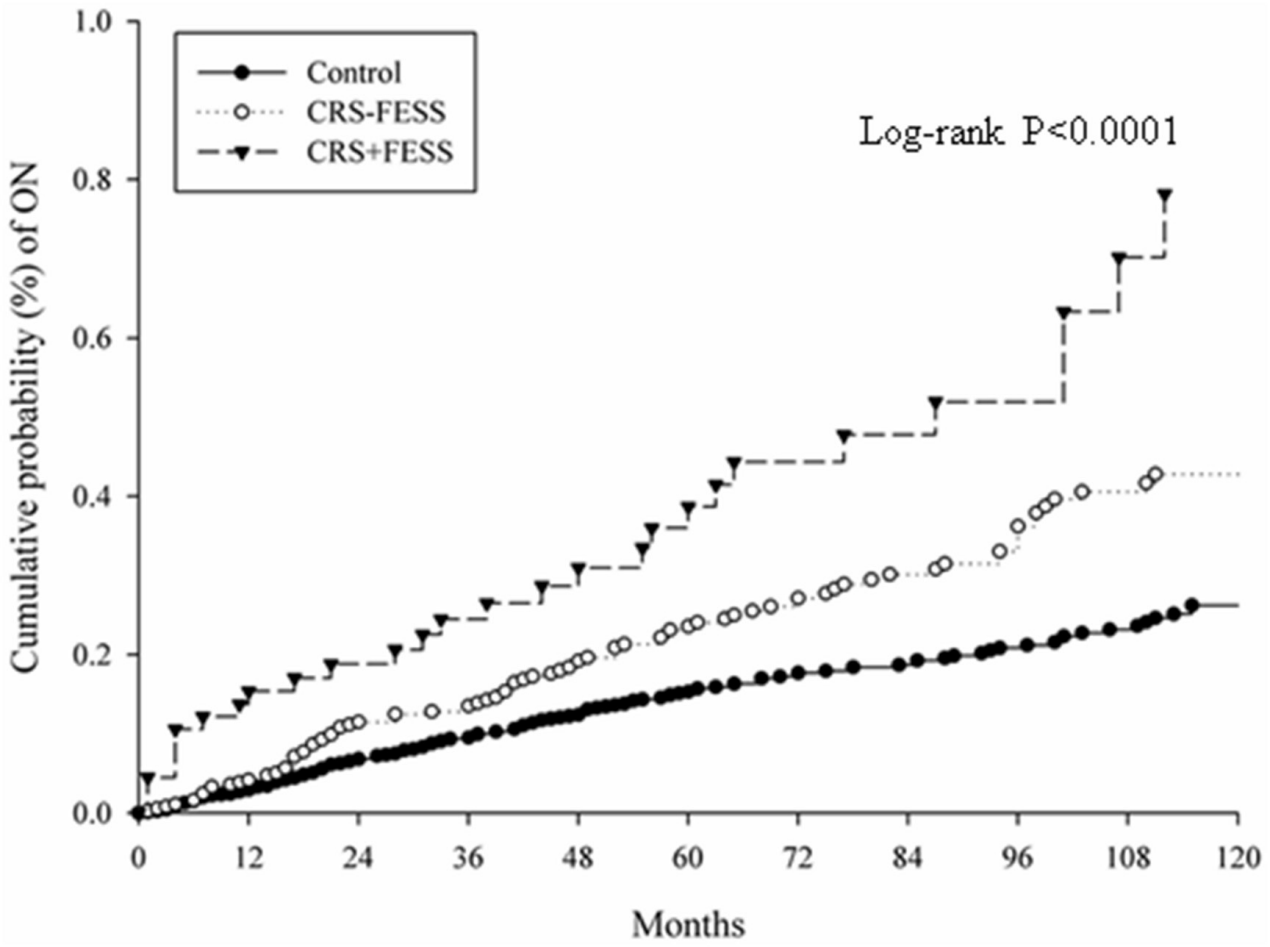
Kaplan-Meier curves of cumulative probability for optic neuropathy. CRS: chronic rhinosinusitis, FESS: Functional endoscopic sinus surgery.

There were 131 and 144 events of ON occurred in the study group and the control group respectively during the whole follow-up period. The study group, regardless of treatment with FESS or not, demonstrated a significantly higher incidence rate, crude RR, and crude HR compared to the control group (Table 2). Multivariable analysis after stepwise selection in multivariable Poisson model showed a significantly higher aRR in both subgroups (Table 3). The CRS without FESS subgroup had an aRR of 1.5 (95% CI: 1.2–2.0), and the CRS with FESS subgroup had an aRR of 2.5 (95% CI: 1.7–3.7) compared to control group. Besides, a significant aHR was also observed in patients diagnosed with CRS but without FESS (aHR: 1.6, 95% CI: 1.2–2.1), and patient diagnosed with CRS and received FESS (aHR: 2.7, 95% CI: 1.5–4.7) compared to control group after stepwise selection in conditional Cox proportional-hazards model (Table 4). Also, the cumulative probability of ON was significantly higher in CRS group (especially in surgery-indicated CRS) than the control group present by Kaplan–Meier curves (Log-rank P<0.0001) (Fig 2).

**Table 2:** Incidence of optic neuropathy in the study and control groups.

	Control n = 88,352	CRS n = 44,176	CRS without FESS n = 37,498	CRS with FESS n = 6,678
Mean / Median of follow up time	71.3/70	71.1/69	71.5/70	68.9/67
Follow up person months	6,301,611	3,142,757	2,682,838	459,919
New ON case	144	131	101	30
Medium Time (Q1 to Q3) from index date to outcome	34(17–61)	40(17–72)	41(18–72)	36(7–65)
Incidence rate*(95% CI)	2.3(1.9–2.7)	4.2(3.1–5.0)	3.8(3.1–4.6)	6.5(4.6–9.3)
Crude RR (95% CI)	Reference	1.8(1.4–2.3)	1.6(1.3–2.1)	2.9(1.9–4.2)
Crude RR (95% CI)	Reference	1.9(1.5–2.4)	1.7(1.3–2.3)	2.8(1.6–4.8)

CRS: chronic rhinosinusitis

FESS: Functional endoscopic sinus surgery

CI: confidential interval

* Incidence rate, per 100,000 person months

RR: relative risk, estimated by Poisson regression

HR: hazard ratio, estimated by Cox regression

**Table 3:** Univariate and multivariable Poisson regression for estimation of crude and adjusted relative risk of neuropathy.

Variable	Crude RR (95% CI)	aRR in full model (95% CI)	aRR in stepwise selection (95% CI)
CRS (Reference: Control)	1.8(1.4–2.3)		
CRS without FESS	1.6(1.3–2.1)	1.4(1.1–1.8)	1.5(1.2–2.0)
CRS with FESS	2.9(1.9–4.2)	2.4(1.6–3.6)	2.5(1.7–3.7)
Age (Reference:40–59)			
<40	0.4(0.3–0.6)	0.5(0.4–0.8)	0.5(0.3–0.7)
60–79	2.6(2.0–3.4)	1.9(1.4–2.5)	2.1(1.6–2.8)
>= 80	3.9(2.3–6.4)	2.3(1.3–4.1)	2.9(1.7–4.9)
Sex (Reference: Female)			
Male	1.2(0.9–1.5)	1.3(0.9–1.7)	1.3(0.9–1.7)
Co-morbidities			
Hypertension	3.5(2.8–4.5)	1.2 (0.9–1.7)	
Diabetes mellitus	3.2(2.4–4.1)	1.2(0.9–1.7)	
Ischemic heart disease	3.0(2.3–4.0)	0.9(0.6–1.3)	
Hyperlipidemia	3.0(2.3–3.8)	1.2(0.9–1.6)	1.4(1.1–1.9)
Heart failure	3.8(2.6–5.6)	1.3(0.8–1.9)	1.4(0.9–2.2)
Cerebrovascular disease	3.9(2.9–5.2)	1.3(0.9–1.9)	1.6(1.1–2.2)
Dementia	2.3(0.9–6.1)	0.5(0.2–1.5)	0.6(0.2–1.6)
Chronic pulmonary diseases	2.2(1.7–2.7)	1.2(0.9–1.6)	
Rheumatic disease	2.7(1.6–4.7)	1.4(0.8–2.3)	
Hemiplegia or paraplegia	4.3(2.2–8.4)	1.8(0.9–3.7)	
Glaucoma	2.5(1.8–3.5)	1.6(1.2–2.3)	1.8(1.3–2.5)
AMD	4.3(2.2–8.8)	1.5(0.6–3.6)	
Retinal vessel occlusion	8.4(3.2–22.5)	2.7(1.0–7.4)	
Posterior and pan-uveitis	11.2(3.6–34.9)	3.9(0.9–16.6)	

aRR = adjusted relative risk

CI = confidential interval

CRS: chronic rhinosinusitis

FESS: Functional endoscopic sinus surgery

AMD = age-related macular degeneration

**Table 4:** Multiple Cox proportional hazard regression for estimation of adjusted hazard ratios on optic neuropathy.

Variable	Crude HR (95% CI)	aHR in full model (95% CI)	aHR in stepwise selection (95% CI)
CRS(Reference: Control)	1.9(1.5–2.4)		
CRS without FESS	1.7(1.3–2.3)	1.5(1.1–2.0)	1.6(1.2–2.1)
CRS with FESS	2.8(1.5–4.8)	2.4(1.3–4.4)	2.7(1.5–4.7)
Age (Reference:40–59)			
<40	-	-	
60–79	-	-	
>= 80	-	-	
Sex (Reference: Female)			
Male	-	-	
Co-morbidities			
Hypertension	1.6(1.2–2.3)	1.2(0.8–1.7)	
Diabetes mellitus	1.6(1.1–2.2)	1.1(0.7–1.7)	
Ischemic heart disease	1.3(0.9–1.9)	0.8(0.5–1.2)	
Hyperlipidemia	1.9(1.4–2.7)	1.6(1.1–2.5)	1.8(1.2–2.6)
Heart failure	2.3(1.3–4.1)	2.1(1.1–4.1)	2.3(1.2–4.2)
Cerebrovascular disease	1.5 (1.0–2.3)	1.4(0.9–2.4)	1.5(0.9–2.4)
Dementia	0.5(0.1–1.7)	0.3(0.1–1.2)	0.2(0.1–0.9)
Chronic pulmonary diseases	1.7(1.2–2.4)	1.2(0.8–1.7)	
Rheumatic disease	2.4(1.1–5.3)	1.4(0.6–3.5)	
Hemiplegia or paraplegia	1.7 (0.7–4.4)	1.2(0.4–4.0)	
Glaucoma	1.9 (1.2–3.0)	1.7(1.0–2.8)	1.8(1.1–2.9)
AMD	1.2(0.5–2.8)	0.8(0.2–2.8)	
Retinal vessel occlusion	1.6(0.4–6.0)	1.0(0.2–4.3)	
Posterior and pan-uveitis	3.0(0.5–17.9)	2.4(0.3–23.0)	

aHR = adjusted hazard ratio

CI = confidential interval

CRS: chronic rhinosinusitis

FESS: Functional endoscopic sinus surgery

AMD = age-related macular degeneration

Of all other potential risk factors of ON included in the multivariable model, aRR and aHR of hyperlipidemia and glaucoma were found to be significant (Tables 3 and 4). In the subgroup analysis stratified by age and gender, the female gender with surgery-indicated CRS and patient with CRS aged younger than 40 exhibited a significantly higher aHR of developing ON (Table 5).

**Table 5:** The sensitivity analysis for the adjusted hazard ratio stratified by gender and age groups.

Subgroups	Incidence rate (95% CI) of ON				
	Control	CRS without FESS	CRS with FESS	aHR 1 (95% CI)	aHR 2 (95% CI)
**Gender subgroups**					
Male	2.5(2.0–3.1)	4.6(3.6–5.9)	4.6(2.765–7.9)	1.9(1.3–2.7)	1.6(0.7–3.5)
Female	2.1(1.7–2.7)	2.9(2.1–4.0)	9.7(6.0–15.6)	1.3(0.8–1.9)	5.8(2.2–15.3)
**Age at index date**					
<40	0.7(0.4–1.0)	2.0(1.3–2.9)	3.5(1.6–7.6)	3.3(1.7–6.4)	10.1(1.1–94.1)
40–59	2.3(1.7–3.0)	3.4(2.4–4.9)	6.4(3.7–11.0)	1.4(0.8–2.4)	3.2(1.2–8.4)
60–79	6.1(4.8–7.8)	9.3(6.8–12.6)	12.3(6.7–22.8)	1.6(1.0–2.4)	1.8(0.7–4.6)
>= 80	11.1(6.3–19.5)	9.5(4.0–22.9)	30.7(4.3–217.7)	1.0(0.2–3.9)	1.3(0.1–23.7)

ON: optic neuropathy

CI: confidential interval

CRS: chronic rhinosinusitis

FESS: Functional endoscopic sinus surgery

aHR: adjusted hazard ratio, adjusted for Hyperlipidemia, Heart failure, Cerebrovascular disease, Dementia and glaucoma in conditional Cox regression

aHR1: CRS without FESS compared to control

aHR2: CRS with FESS compared to control

## Discussion

In the current study, we demonstrate a significant association between ON and CRS after adjusting for multiple potential risk factors. In addition, the possibility of occurring ON is also prominently elevated with the existence of hyperlipidemia and glaucoma.

Several possible mechanisms of CRS may be related to the development of ON. The nasal sinus, especially the sphenoid and ethmoidal sinuses, are adjunct to the orbital apex where the optic nerve is located. As a result, the inflammatory or infectious lesions of CRS may invade and influence the nearby tissue and contribute to orbital apex syndrome and damage of optic nerve.[20, 21] Besides, the swelling of paranasal sinus in patients with CRS can also lead to compressive injury of optic nerve and following compressive ON as revealed in previous studies.[18, 22] In addition, the inflammatory process accounts for the majority of etiology for ON including the viral, autoimmune-related or idiopathic subtypes.[23] The elevated inflammatory mediators including interleukin and IgE in the CRS might elevate the local inflammatory reaction,[24–26] then lead to the occurrence or progression of ON. In summary, the above evidence supports the hypothesis that an event of CRS may contribute to the development of certain types of ON, which corroborates with our findings.

In the current study, the patients with both surgery-indicated CRS and CRS without surgical management owned a significantly higher possibility to develop ON compared to non-CRS individuals with a higher cumulative probability. Moreover, we excluded patients with pre-existing ON to prevent mis-calculation of outcome achievement. To our knowledge, this is a preliminary experience to demonstrate the casual relationship between ON and CRS with an adequate length of follow-up period. It also demonstrated an even high risk for those with severe CRS requiring FESS. Considering previous research to reveal the development of ON in patients with CRS with or without the performance of FESS, decreased retinal nerves fiber layers and ganglion cell complex thickness were found in patients with several types of CRS.[19] Despite the phenomenon was observed,[19] the case numbers of CRS were few with only 103 patients in that study and the casual relationship between ON and CRS remained not fully elucidated due to the cross-sectional nature.[19] In our study, however, the population-based design included adequate case numbers, and the multivariable analysis showed CRS need FESS to treat was at highest to develop ON which not be shown in previous study. On the other hand, nearly all patients with CRS were treated with corticosteroid which can also be used to manage ON,[8, 27, 28] but the significant results in the current study imply that the effect of both surgery-indicated CRS and non-surgery CRS overwhelms the therapeutic effect of steroid on the ON.

The incident rate of ON is higher in the female population than the male population with surgery-indicated CRS in the current study. Generally, the female is more vulnerable to the ON than the male population according to previous study.[29, 30] In one population-based study, the incidence of ON in female is 1.67 folds higher than male.[31] The results concerning the effect of female gender on the development of ON in those with surgery-indicated CRS in the current study is corresponded to the previous experience, while those with CRS but without surgery management showed conflicting results. Further studies are required to determine if the higher ratio of ON in female population with surgery-indicated CRS was attributed to the female gender or to disease severity.

About other diseases that correlated to the occurrence of ON, glaucoma is the ocular disease with a significantly higher aRR and aHR. It is reasonable for this correlation since glaucoma itself is one type of progressive optic neuropathy that present with visual field defect, and persistent glaucoma may lead to degeneration of optic nerve head.[32] In addition, both the AMD and retinal vessel occlusion-induced ischemia are associated with nerve damage,[33, 34] in which the two diseases revealed an marginally elevated risks for developing ON in the current study after multivariable analysis. The pathophysiology of hyperlipidemia as a risk factor of ON needs further evaluation. Besides, although ON commonly occurs in population with young to middle age,[35] the age was matched in the current study so the influence of age on the relationship between surgery-indicated CRS and ON might be neglected.

The CRS affected about one percent of population in Taiwan according to a previous study conducted in the same region.[36] In the current study, the surgery-indicated CRS account for about 0.3 percent in all population. The lower occurrence rate in the current study compared to the previous study may due to we only enrolled those patients with severe CRS that need FESS to manage. Concerning the epidemiology of ON, the percentage of ON in the control group of current study is similar to the general population in other epidemiological studies of Taiwan with an occurrence rate about 0.1 percent.[31, 37] However, the occurrence rate of ON was near 0.4 percent in the study group, which was significantly higher than the occurrence rate in the control group according to the multivariable analysis and the occurrence rate in the previous studies conducted in the same population.[31] The 2-folds higher occurrence rate of ON of the study group in the current study further strengthened the universality of our findings and illustrated the clinical importance of surgery-indicated CRS on the optic nerve.

There are some limitations in the current study. First, the observational and retrospective nature of study design may reduce the homogeneity of patient population even using multivariable analysis. In addition, we used claimed data rather than real medical documents to analyze, thus missing some important information like the laterality and severity of ON and the postoperative condition of CRS after FESS procedure, and some patients with ON may also be missed since we did not assess the patient directly. Moreover, there are different types of ON (i.e. autoimmune-related, viral infection and idiopathic form), thus we cannot decide which subtype of ON resulting from the influence of CRS which make the causal-relationship between the two diseases unclear. But since many types of ON shares the inflammatory nature,[38] the surgery-indicated CRS might own influence in many types of ON due to its inflammatory reaction and a universal relationship between these two diseases may exist.

In conclusion, the existence of CRS is a significant risk factor in developing ON. Furthermore, the risk of developing ON is positively elevated especially in those patients with surgery-indicated CRS who received FESS management. Further large-scale study to reveal the effectiveness of both non-surgery and surgery-indicated CRS on different subtypes of ON is mandatory.

## Supporting information

S1 Fig The variation of proportional-hazards depended on follow up time by using the log-log plot. (DOCX)

S1 Table List of codes for co-morbidities. (DOCX)

S2 Table The code for univariate Poisson regression. (DOCX)
