# Supplementary material for: The development of optic neuropathy after chronic rhinosinusitis: A population-based cohort study
Source: PLoS One. 2019 Aug 7;14(8):e0220286. doi: 10.1371/journal.pone.0220286 (PMC6685625; doi:10.1371/journal.pone.0220286)
Supplement: S1 Fig — (DOCX) [file pone.0220286.s001.docx]

**S1 Fig**

**
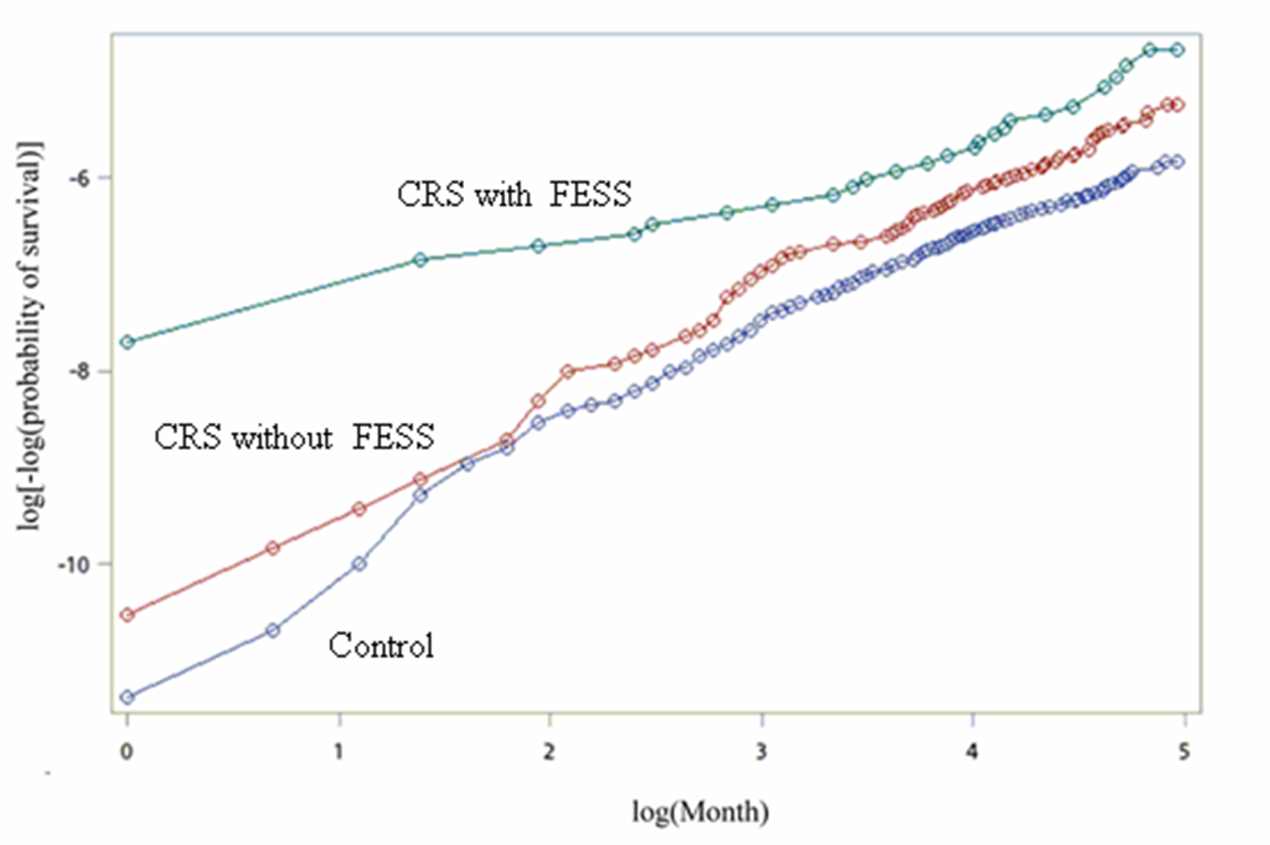
**

**S1 Fig.** The variation of proportional-hazards depended on follow up time by using the log-log plot.

CRS: chronic rhinosinusitis

FESS: Functional endoscopic sinus surgery
