# Supplementary material for: The development of optic neuropathy after chronic rhinosinusitis: A population-based cohort study
Source: PLoS One. 2019 Aug 7;14(8):e0220286. doi: 10.1371/journal.pone.0220286 (PMC6685625; doi:10.1371/journal.pone.0220286)
Supplement: S1 Table — (DOCX) [file pone.0220286.s002.docx]

**S1 Table**

List of codes for co-morbidities

Hypertension (ICD-9 codes: 401.x-405.x, ICD-10 codes: I10, I11.x, I13.x, I15.x, I16.x, I87.3x, I97.3x, O10.x, O11.x, O13.x, O16.x)

Diabetes mellitus (ICD-9 codes: 250.x, 277.7, ICD-10 codes: O24.4, E11.x, E13.x, E88.81)

Ischemic heart diseases (ICD-9 codes: 410.x, ,412.x, 414.0, 414.0x, 414.2, 414.3, 414.4, 414.8, 414.9, ICD-10 codes: I20.x-I25.x)

Hyperlipidemia (ICD-9 codes: 272.0, 272.1, 272.2, 272.4, 272.9, ICD-10 codes: E78.0x, E78.1, E78.2, E78.3, E78.4x, E78.5, E78.70, E78.79, E78.89, E78.9)

Congestive heart failure (ICD-9 codes: 398.91, 402.01, 402.11, 402.91, 404.01, 404.03, 404.11, 404.13, 404.91, 404.93, 425.4–425.9, 428.x, ICD-10 codes: I50.2x, I50.3x, I50.4x, I50.84, I50.89, I50.9)

Cerebrovascular disease (ICD-9 codes: 362.34, 430.x–438.x, ICD-10 codes: G46.x, I60.x-I66.x, I67.0, I67.1, I67.2, I67.6, I67.81, I67.82, I67.84x, I67.89, I67.9)

Dementia (ICD-9 codes 290.x, 294.1, 331.2, ICD-10 codes: F01.x, F02.x, F03.x, G31.x)

Chronic pulmonary disease including asthma (ICD-9 codes: 416.8, 416.9, 490.x–505.x, 506.4, 508.1, 508.8, ICD-10 codes: J41.x, J42, J43.x, J44.x, J47.x)

Rheumatic disease (ICD-9 codes: 446.5, 710.0, 710.1, 710.3, 710.4, 714.0–714.2, 714.8, 725.x, ICD-10 codes: M05.1x, M05.2x-M05.9, M31.6, M32.1x, M32.8, M32.9, M33.03, M33.13, M33.2x, M33.90, M33.93, M34.0x, M34.1x, M34.9, M35.3)

Hemiplegia or paraplegia (ICD-9 codes: 334.1, 342.x, 343.x, 344.0–344.6, 344.9, ICD-10 codes: G80.0-G80.2, G80.8, G80.9, G81.x, I69.33x-I69.36x, I69.83x-I69.86x, I69.93x- I69.96x)

Glaucoma (ICD-9 codes: 365.1x, 365.2x, 365.7x, 365.9, ICD-10 codes: H40.1x, H40.2x, H40.89, H40.9)

Retinal vessel occlusion (ICD-9 codes: 362.3x, ICD-10 codes: H34.x)

Age-related macular degeneration (ICD-9 codes: 362.50, 362.51, 362.52, ICD-10 codes: H35.30, H35.31x, H35.32x)

Posterior as well as pan-uveitis (ICD-9 code:360.12, 363.0x, 363.1x, 363.20, 363.22, ICD-10 code: H30.0x, H30.1x, H30.8x, H30.9x, H44.11x)
