# Supplementary material for: The development of optic neuropathy after chronic rhinosinusitis: A population-based cohort study
Source: PLoS One. 2019 Aug 7;14(8):e0220286. doi: 10.1371/journal.pone.0220286 (PMC6685625; doi:10.1371/journal.pone.0220286)
Supplement: S2 Table — (DOCX) [file pone.0220286.s003.docx]

**S2 Table**

The code for univariate Poisson Regression was listed below:

"

proc genmod data=research_1;

class exposure;

model count= exposure / offset=logt dist=poisson

link=log;

run;
